# The Role of Humor in Inoculation Theory

**DOI:** 10.3390/bs16040502

**Published:** 2026-03-27

**Authors:** Josh Compton, Sander van der Linden

**Affiliations:** 1 Speech at Dartmouth, Dartmouth College, Hanover, NH 03755, USA; josh.compton@dartmouth.edu; 2 Department of Psychology, Downing Site, University of Cambridge, Cambridge CB2 3EB, UK

**Keywords:** inoculation theory, humor, resistance to persuasion, misinformation

## Abstract

Inoculation is serious business, but that does not mean it cannot be viewed through a perspective of humor, nor that humor cannot be part of the process of attitude resistance. This applies to both medical and communication-based inoculation, with scientists examining how humor could function as an adjuvant and facilitate resistance to persuasive attacks. Though relatively limited, considerations of humor have been part of inoculation theory from the beginning—dating back from McGuire’s seminal 1964 review to the prominent role of humor in modern gamified inoculation interventions against misinformation. In this article, we explore humor’s role in inoculation theory, review emerging research, and propose promising directions for future theory and application.

## 1. Introduction

In both medicine and communication, inoculation addresses matters of life and consequence. Medical inoculation protects against some of the world’s most serious viral threats—smallpox, measles, polio, and more (Montero et al., 2024). Psychological inoculation protects against some of the world’s most serious communication threats—mis- and disinformation, propaganda, radicalization, and more (Compton, 2025; van der Linden, 2023, 2024). As Compton (2019) put it, “With both types of inoculations, stakes are high” (p. 1). At the same time, even with so much on the line, and with the severity of harms inoculation seeks to mitigate—in both medicine and communication—there is room for humor. The person who first named and explained inoculation theory, McGuire (1964), opens his seminal 1964 review with a nod to the satirist Molière, and the most recent work on inoculation theory extends it to comics, games, and jokes. But to put a finer point on it, our position is not only that humor can work with inoculation-based interventions, but that it often *should*.

Health professionals have long realized the promise of humor. Medical research has found that humor can both lower stress and activate the immune system, including boosting natural killer (NK) cell activity, immunoglobulins (IgG, IgA, IgM), and T- and B-cell activation (Berk et al., 2001). Although findings remain tentative (Bennett & Lengacher, 2009), the study of humor and medicine continues to guide medical research (e.g., Yamakoshi et al., 2025) and practice (e.g., Rizzolo & Gray, 2025), including longstanding work with medical clowning (e.g., Khoury et al., 2025).

Communication and social psychology scholars have long realized the promise of humor and health, too. Such research has focused on the effects of humor communication on physical health (e.g., Lockwood & Yoshimura, 2014), mental health (e.g., Wagner & Temmann, 2025), and healthy behaviors and behavioral intentions (e.g., walking: Symons et al., 2024; breast self-examination: Liu & Chen, 2024; vaccination intentions: Han et al., 2024). As with many dimensions of inoculation theory, the analogic extension is ripe for scrutiny, especially when we consider the overlap between research on humor and health from both medical and social scientific approaches. In medicine and in the social sciences, humor has been part of the immunity story, and we think it should be part of inoculation theory’s story moving forward, too. Specifically, humor can act as an adjuvant: just as biological adjuvants enhance antigen delivery in medical vaccines (CDC, 2024), psychological adjuvants can increase mental antibody production by increasing both cognitive and motivational engagement, two key mechanisms underlying inoculation theory—which we will discuss next.

## 2. Inoculation Theory

Inoculation theory lives up to its name. Just as a body can be made more resistant to future viral attacks through exposure to weakened forms of those attacks (e.g., an attenuated virus used in immunization to jumpstart the body’s defenses), the mind can be made more resistant to future challenges through exposure to weakened forms of those challenges (e.g., a two-sided message used in a communication intervention to jumpstart the mind’s defenses; see McGuire, 1964; Ivanov et al., 2026). Core to both types of inoculation—medical and psychological—is threat, the recognition that a position is vulnerable to influence (Compton, 2021; McGuire, 1964). It is not that more threat necessarily equals more resistance—Banas and Rains’ (2010) meta-analysis clarified that—but that threat might be better understood as a threshold concept (Compton, 2025), where enough threat is elicited by the inoculation intervention to trigger resistance processes, distinguishing it from other interventions, like literacy training (Kuru, 2025).

A common way to elicit threat in a conventional inoculation intervention is with a forewarning—a message that usually precedes any refutational content—that lets people know that their position will be challenged (Compton, 2025; McGuire, 1964). But while forewarnings are a common way to generate threat in an inoculation-based intervention against influence, forewarnings are not the only way to generate threat. The mere presence of counter-content, like counterarguments raised and refuted in a two-sided message format, or exposure to misinformation strategies, can also elicit threat (Compton, 2025; McGuire, 1964; Basol et al., 2021). Some research suggests that when more than one threat-eliciting component is included in an inoculation intervention, the forewarning is most responsible for eliciting threat (Compton & Ivanov, 2012).

The goal of an inoculation intervention does not end with threat, of course. Inoculation’s success means more resistance to influence—the protection of a position against a challenge or attack. In McGuire’s earliest work, position meant a belief about an issue, like the safety of X-rays or the importance of teeth brushing (McGuire, 1964). Later, scholars working with inoculation would extend the scope to attitudes (e.g., Pfau & Burgoon, 1988), and later, to behavioral intentions (e.g., intentions to drink alcohol, Godbold & Pfau, 2000), emotional states (e.g., public speaking anxiety, Jackson et al., 2017), misinformation strategies (Compton et al., 2021; van der Linden, 2024; van der Linden & Roozenbeek, 2021) and psychological responses (e.g., reactance, Clayton et al., 2023). Because inoculation has established efficacy beyond beliefs to include these other components, we will use the term *position* here to stand for any state that can be protected against change.

Before we consider how humor might function in and around inoculation theory, it is important to clarify three important things about inoculation theory. First, although the earliest research applied inoculation messaging to non-controversial topics or what McGuire (1964) termed ‘cultural truisms’ (meaning beliefs that were so well established, so common, that one would not likely have considered counter-positions), inoculation theory researchers have extended the scope to include controversial, contested issues soon after. By the time inoculation theory research hit its resurgence in the late 1980s, much inoculation work was exploring contested, controversial issues, including issues of politics, health, and commerce (see Compton & Pfau, 2005); though most research remained unconcerned with the context that originally motivated inoculation theory: propaganda (van der Linden, 2023, 2024). Accordingly, during inoculation’s latest resurgence, inoculation has been applied to some of the most contested issues, including vaccine safety and effectiveness, climate, and conspiracy theories (see Banas et al., 2024; Compton et al., 2021).

Second, in the first decades of inoculation theory work, inoculation was conceptualized as an intrapersonal process—how inoculation-based interventions change how individuals think (McGuire, 1964). But more recent inoculation research has confirmed that inoculation is an interpersonal process, too. For example, inoculation interventions lead to more talk about the issues through post-inoculation talk (Compton & Pfau, 2009), which could spread the inoculation across social networks and beyond, leading to a type of herd immunity (van der Linden, 2024). Finally, although most inoculation theory research in the past 60+ years has treated inoculation as a prophylactic, or preemptive strategy (i.e., protecting a position before it is attacked), more recent work has studied and confirmed therapeutic applications of inoculation (see Compton, 2020), or inoculations administered without the “right” position already in place (e.g., van der Linden et al., 2017). Overall, meta-analyses have established the efficacy of inoculation as a robust defense against influence and (malicious) persuasion (Banas & Rains, 2010; Simchon et al., 2025).

## 3. Humor in Inoculation Theory

**Figure 1:**
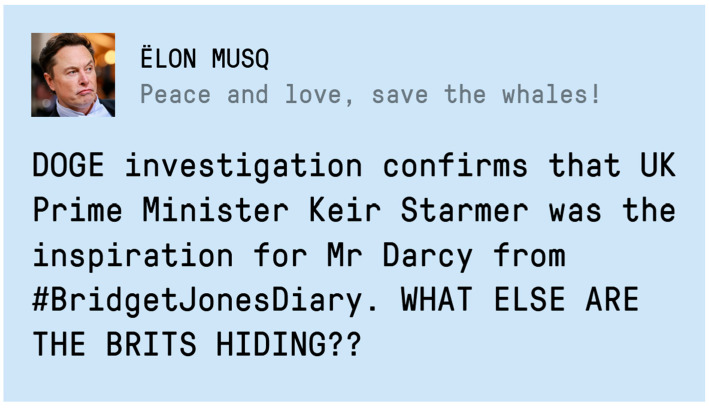
Humorous example from the *Bad News* game. Adopted with permission from van der Linden (2023). *Note*: Trolling tactic illustrated with a weakened-dose example using Elon Musk for comedic effect.

**Figure 2:**
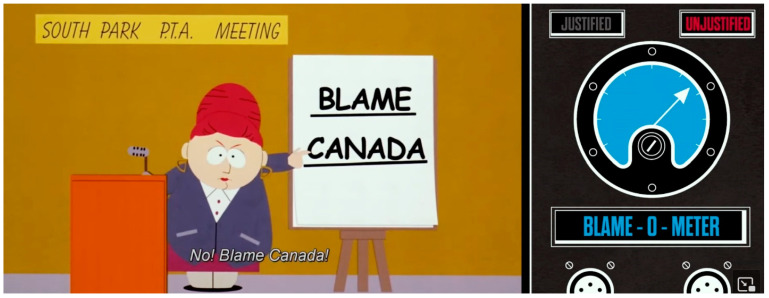
Humorous weakened-dose example from inoculation video against scapegoating using Southpark episode (adopted with permission from Roozenbeek et al., 2022). *Note*: In the video, “the kids are getting worse” disobedience problem is unfairly blamed on Canada.

With some inoculation theory and humor research, the humor is right there in the treatment—a joke, a comic, a pun. This is the type of relationship between humor and inoculation theory we will explore in this section—humor used *in* inoculation treatments. Humor has a long history in influence and persuasion research (Lull, 1940; Limbu et al., 2026; Nabi et al., 2007; Walter et al., 2018; Young, 2020) and studies have found that humor can reduce psychological reactance on controversial topics such as vaccination (Moyer-Gusé et al., 2018) and lead to more positive affect and greater knowledge acquisition (Walter et al., 2018; Limbu et al., 2026). Some inoculation research on controversial topics such as climate change has explored the role of humor. In one study, participants were inoculated against a fake petition claiming many scientists have declared global warming a hoax—here the weakened-dose refutation consisted of humorous examples such as signatories named ‘Dr Geri Halliwell’ from the Spice Girls or ‘Charles Darwin’ (van der Linden et al., 2017). Even more explicitly, gamified inoculation against misinformation has incorporated humor into the design of technique-based inoculation interventions (technique- or logic-based inoculations focus on the underlying tricks and fallacies rather than specific issues, see Banas & Miller, 2013). For example, the *Bad News* game offers a simulated social media feed where players are forewarned against the threat of misinformation and exposed to weakened doses of manipulative and propagandistic strategies (Traberg et al., 2022; Roozenbeek & van der Linden, 2019). Many of the ‘weakened-dose’ strategies in the game explicitly rely on sarcasm, satire, jokes about elites, and self-deprecating humor in order to lower people’s defenses on the topic of misinformation. For example, in order to illustrate ‘trolling’, the game leverages a comedic example from Elon Musk (Figure 1) where he trolls the UK Prime Minister Sir Keir Starmer by suggesting he was the inspiration for Mr. Darcy from the popular movie Bridget Jones’ Diary. This is fake, but given Musk’s frequent attacks on the UK prime minister this exchange generates a comedic effect by illustrating a weakened dose of a trolling attack. In a real-world inoculation campaign with Google, inoculation video scripts were developed to forewarn audiences on YouTube about the dangers of online manipulation along with animated versions of the weakened dose. For example, one video inoculated audiences against unfair scapegoating, where the ‘weakened dose’ is a short clip from Southpark (Figure 2) where they decide to blame ‘Canada’ for all societal ills (Roozenbeek et al., 2022). Many inoculation-inspired games have adopted a humorous approach to prebunk misinformation, including the *Cranky Uncle* cartoon which leverages the stereotype of an old cranky uncle who relies on logical fallacies to deny climate change as a way to induce resistance to climate disinformation (Cook, 2020; Cook et al., 2023).

Inoculating against misinformation, then, often turns to humor, and as Petty (2024) has argued, the lessons learned about resistance to misinformation can inform research on attitudes more generally, even when the domain is less true–false and more opinion-oriented. Consider, for example, that Warner et al. (2015) found that watching a political parody segment prior to exposure to a Super PAC political advertisement mitigated the persuasiveness of the political ad—an effect more on attitudinal change than on misinformation claims (and see Compton, 2018, for a review of inoculation theory and political humor). Importantly, the meaning and impact of humor varies across individuals and cultures (J. G. Lu, 2023) and even the political spectrum with conservatives and liberals revealing different preferences for specific types of humor (Kfrerer et al., 2021; Wilson, 1990; Young, 2020). For example, conservatives often perceive late night comedy as having a strong liberal bias (Dagnes, 2012) which has ramifications for how the use of humor in inoculation might be received by different audiences (e.g., inoculations that use late night comedy or satire might work less well for conservative versus liberal audiences).

These studies also raise the question of how humor is operationalized as an adjuvant in psychological inoculation research. Whether presented as jokes, games, or comics, the most common approach seems to be to use humor as a way to discount (misinformation) claims and weaken or dilute the dose in the eyes of the participants. However, we should note that alternative approaches should be explored as well, for example, humor could be used to elicit motivational threat or lower psychological reactance (Richards et al., 2017). Other research has looked at humor not as an inoculation treatment, but as a sort of “booster shot” accompanying conventional inoculation interventions. For example, Compton (2004) found that a candidate appearing on a late-night television talk show could boost the inoculative effects of a conventional written inoculation message (a message that forewarned against impending challenges and preemptively raised and refuted some of those challenges) against a conventional political attack (a written essay criticizing that candidate). These efforts situate humor as part of the conversation of “boosters” in inoculation theory more generally (see Banas & Bessarabova, 2026; Matig, 2026).

## 4. Humor and Inoculation Theory

Even when inoculation-based interventions do not explicitly use humor—a joke as part of the preemptive refutation, a funny comic accompanying an explanation of a reasoning fallacy—humor can still be part of inoculation theory’s story. We turn to this relationship between humor and inoculation theory here—humor *and* inoculation theory.

Consider, for example, how humor can be part of the challenge to be inoculated against. Compton’s (2004) early inoculation work looked at that—how to inoculate against political attacks that come in the form of late-night television political humor, like monologue jokes poking fun at politicians (think *The Tonight Show*) or satirical sketches and impersonations (think *Saturday Night Live*). Results indicated that a conventional inoculation treatment—a written message that forewarned of the impending political attacks and raised and refuted some of them—failed to confer resistance to attacks embedded in political humor. Not only that, but in some instances, trying to inoculate against political humor made it worse, backfiring against the candidate. Compton reasoned that people might have been surprised to be warned about something as seemingly trivial as late-night political comedy, and so they reconsidered their support for the candidate. Trying to inoculate against late-night television humor did not fare much better; trying to inoculate against the influence of late-night television humor in general failed to protect against its political humor.

Or consider Lim and Ki’s (2007) study of inoculating against YouTube parody videos made by corporate front groups to affect attitudes toward policy positions—in their study, a video spoofing UFO movies about Internet policy. Warning people about ethically suspicious video—preemptively explaining how parody works and raising and refuting some issue-specific counterarguments—led to more resistance to the arguments in the parody videos, identification of the manipulative intent, and it also led to source derogation of the sponsor of the video when compared to those who did not receive an inoculation message.

Then there are Clyne et al.’s (2020) findings that one can inoculate against a specific type of humor used as a communication strategy—in their study, sarcasm. The issue they worked with was mandatory college exit exams, with some persuasive messages using literal approaches and others using sarcasm. Results of their study indicated that “forewarning of the psychological process used by sarcasm eliminated the persuasive advantage of sarcasm in an attack message” (Clyne et al., 2020, p. 77). They also found that, when one is warned about a sarcastic persuasive attempt, but the persuasive attempt is literal—that is, does not contain the sarcasm warned about—it does more harm than good, leading to less resistance. In summary then, humor can be used as part of the inoculation process itself but also as an object to be inoculated *against* (see also Barbati, 2026).

## 5. New Directions for Humor and/in Inoculation Theory

### 5.1. Humor, Inoculation, and the Need for Conceptual and Empirical Validation

The observant reader might wonder how the use of humor fits with the inoculation analogy. Consistent with Compton (2013), we view the inoculation analogy as instructive rather than restrictive. For example, recent advances have included the role of *feedback*. In the body, *t*-cells benefit from a process of trial and error (i.e., feedback) so that they learn to distinguish foreign invaders from healthy cells, so too, does the human mind benefit from an inoculation that uses feedback to help people discern better between manipulation and non-manipulation (Leder et al., 2024).

**Figure 3:**
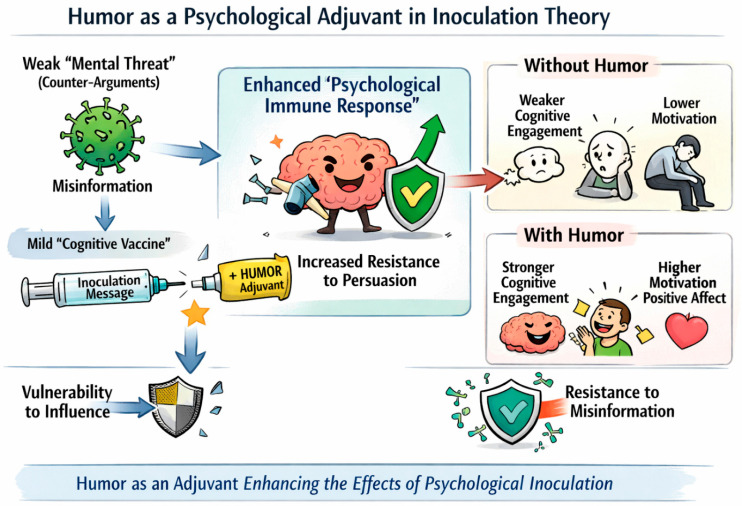
Humor as a psychological adjuvant in inoculation theory. *Note*. A humor-based illustration of some hypotheses about how humor might act as a psychological adjuvant in inoculation interventions.

In the case of humor, we can think of it both as a way to synthesize a weakened dose of an impending attack as well as an *adjuvant* (Figure 3). In the context of immunization, adjuvants are additives that enhance the body’s immune response to an antigen (Reed et al., 2013). In a similar way, humor is thought to behave much like an “adjuvant” in psychological inoculation interventions, maximizing their uptake among potential target audiences. However, so far, the role of humor has seen limited evaluation in inoculation research (see Barbati, 2026). For example, although inoculation-inspired games such as *Bad News* and *Cranky Uncle* have proven efficacious in meta-analyses in terms of their inoculative effects (C. Lu et al., 2023; Simchon et al., 2025), the role of humor has not been tested explicitly either as a main effect, manipulated factor, mediator, or moderator of intervention success. This presents an open area for both basic and applied research. For example, future research could measure the extent to which interventions elicit laughter or humor and whether that acts as an “adjuvant” mechanism predicting intervention efficacy and longevity. Specifically, the mechanisms behind inoculation represent both motivation and ability (McGuire, 1964; van der Linden, 2023). As such, humor might impact both stronger cognitive engagement as well as higher motivation to resist persuasive attacks (Figure 3). Alternatively, games or videos could be produced in such a way that they are matched identically except for the inclusion/exclusion of humor in order to causally evaluate the benefit (or drawbacks) of humor to inoculation outcomes. Humor could be operationalized as a manipulated factor (as above) or measured post-treatment by asking participants how funny or amusing they found the intervention, which could also add new insights about the role of (positive) affect in inoculation research (Pfau et al., 2009). The impact of the inoculation intervention on relevant outcome measures such as attitudinal resistance could be tested with a moderation hypothesis (Figure 3) where one expectation could be that humorous versus non-humorous treatments would elicit stronger resistance. A mechanistic account can be tested by examining the impact of humor on participants’ ability to counter-argue potential attacks, memorize the inoculation material, or increase in perceived motivational threat from the inoculation message.

### 5.2. Humor, Inoculation, and Therapeutic Versus Prophylactic Uses

Increasing attention to attitudinal inoculation as a therapeutic (“healing” a position and then protecting it from further influence) in addition to its more classical application as a prophylactic (preempting an attack before it can “infect”; see Compton, 2020) has broadened inoculation’s scope. At the same time, it is important to consider potential differences in inoculating before or after an attack has taken hold. This matters not only for empirical but also for conceptual reasons. For example, Moyer-Gusé et al. (2018) found that humor (in their study, a clip from *The Daily Show*) could help circumvent parents’ reactance to MMR vaccine advocacy when they have false beliefs about MMR vaccine safety and efficacy. And yet, for parents who already had accurate beliefs about MMR vaccine safety and efficacy, a message that did not use humor worked better for bolstering their beliefs. Might humor-based inoculation interventions work better as a therapeutic than as a prophylactic vaccine? In other words, is its primary use to lower reactance and defensiveness among the already exposed or does humor have universal appeal? This is a question worth pursuing.

### 5.3. Humor, Inoculation Theory, and Duration of Effect

One promising finding from medicine: a one-hour humorous video had positive effects on the biological immune system for up to 12 h afterward (Berk et al., 2001). As such, it is possible that humor could sustain psychological inoculation effects longer than traditional inoculation messages, for example, by making the content more accessible and memorable (Blanc & Brigaud, 2014; Maertens et al., 2025). Future studies could employ longitudinal designs to understand whether humor-based inoculation treatments outperform traditional inoculations and the cognitive and emotional mechanisms associated with increased (or decreased) message longevity.

### 5.4. Humor, Laughter, and Inoculation Theory

To this point, we have treated humor as a bit of a monolith, lumping all types of humor (satire, puns, comics) into the concept of humor. We have also given only passing reference to laughter—the physical manifestation of the largely cognitive process of humor (Gonot-Schoupinsky et al., 2020). Gonot-Schoupinsky et al. (2020) put a finer point on it, proposing clarifying definitions of humor and laughter in their scoping review:

Humor is predominantly a cognitive process, often involving perceptions of funniness, occurring alone or socially. It can be created, appreciated, reminisced, arise spontaneously, or be enacted, e.g., clowns, and serves diverse personal development functions including social bonding. It may be induced by a range of emotions, playfulness, and/or laughter, or induce these. It is influenced by motives, circumstances, and cultural and individual differences.

(p. 4)

Laughter is predominantly a physical behavior, occurring alone or socially. It is often used as a form of verbal expression or communication. It can be spontaneous, provoked, or self-induced, and serves diverse personal development functions including social bonding. It may be induced by a range of emotions, playfulness, and/or humor, or induce these. It is influenced by motives, circumstances, and cultural and individual differences.

(pp. 4–5)

Gonot-Schoupinsky et al. (2020) further offer a model of humor and its effects on well-being—the Humor–Laughter–Affect (HuLA) model—that shows myriad possibilities for how different types of humor, different affective responses, and different laughs can interact. This model, and others like it, can help researchers tease out more precise effects of humor (including styles, like cynicism and satire), laughter (including differing valences, like positive and negative laughter), and affect (including positive and negative, like joy and spite). We could imagine, for example, that an inoculation intervention that mocks the source of counterattitudinal messaging could elicit negative affect toward that source in ways that strengthens resistance, and vice versa, that good-natured teasing of the source of a counterattitudinal message might lessen resistance (e.g., Pfau et al., 2001, 2009). Researchers should not stop at the broad concept of humor, but instead, turn to its many types, valences, and interactions, aided by models like HuLA and other humor and entertainment classification systems (e.g., Holbert, 2005).

## 6. Conclusions

Just as inoculation scholars have warned that inoculation is not a fix-all approach to communication threats, including sustained attention to inoculation theory’s boundary conditions (Ivanov, 2017), humor scholars have warned the same, e.g., “it would be unwise to conclude that humor is a magic bullet that simply thwarts biased processing and motivated reasoning” (Moyer-Gusé et al., 2018, p. 519). Miczo (2019) has noted that “communication scholars are a long way from being positioned to offer empirically grounded advice regarding how to use humor to improve people’s lives (without necessarily implying that other disciplines are in any better position)” (p. 14). Nonetheless, we have argued that humor has a place in inoculation theory as a potentially important adjuvant. We hope some of the ideas we have sketched out here can help inform future research on the role of humor in inoculation theory, if not from research that pursues our questions, then from research that asks even better questions than what we have suggested here.

## Data Availability

No new data were created or analyzed in this study.
